# Local Antimicrobial Therapy with Combined Aminoglycoside and Vancomycin Compared to Aminoglycoside Monotherapy in the Surgical Management of Osteomyelitis and Fracture-Related Infection

**DOI:** 10.3390/antibiotics13080703

**Published:** 2024-07-27

**Authors:** Annalise Unsworth, Bernadette Young, Jamie Ferguson, Matthew Scarborough, Martin McNally

**Affiliations:** 1 The Bone Infection Unit, Nuffield Orthopaedic Centre, Oxford University Hospitals, Oxford OX3 7LD, UK; annalise.unsworth@svha.org.au (A.U.); bernadette.young@ouh.nhs.uk (B.Y.); jamie.ferguson@ouh.nhs.uk (J.F.); matthew.scarborough@ouh.nhs.uk (M.S.); 2 St Vincent’s Hospital Clinical School, University of New South Wales, Sydney 2010, Australia

**Keywords:** fracture-related infection, FRI, bone infection, osteomyelitis, local antibiotics, gentamicin, vancomycin

## Abstract

We investigated the effect of combination aminoglycoside and vancomycin local antibiotic treatment compared to aminoglycoside alone in the surgical management of bone infection. Data including patient demographics, type of surgery, microbiological characteristics, BACH score, duration of antibiotic treatment and clinical outcomes were collected. Failure of therapy was a composite of recurrence of infection, continued or new antimicrobial therapy, or reoperation with suspected or confirmed infection at one year after index surgery. A total of 266 patients met the inclusion criteria. 252 patients reached the final follow-up and were included in the final analysis. 113 patients had treatment with aminoglycoside alone and 139 patients had combination aminoglycoside and vancomycin. There was no difference in the failure rate between groups; 10/113 (8.8%) in the aminoglycoside alone and 12/139 (8.6%) in the combination group, *p* = 0.934. Multivariate analysis showed that there was no added benefit of combination therapy (OR 1.54: 95% CI 0.59–4.04, *p* = 0.38). BACH score and low BMI were associated with increased risk of failure (BACH OR 3.49: 95% CI 1.13–10.76, *p* = 0.03; Low BMI OR 0.91: 95% CI 0.84–0.99, *p* = 0.037). The form of the carrier material (pellets or injectable paste) had no effect on failure rate (*p* = 0.163). The presence of aminoglycoside resistance had no effect on failure rate (OR 0.39: 95% CI 0.05–3.01, *p* = 0.37). Clinical outcome was not improved by the addition of vancomycin to aminoglycoside alone as local therapy for the management of bone infection.

## 1. Introduction

Osteomyelitis and fracture-related infection (FRI) cause significant morbidity, often requiring surgical debridement and medical therapy with a combination of local and systemic antibiotic therapy. Local antibiotics delivered directly to the site of infection at the time of surgery achieve high tissue concentrations with limited risk of systemic side effects [1,2,3]. 

Local implantation of aminoglycosides (gentamicin and tobramycin) provides levels well above the minimum inhibitory concentration (MIC) of typical infecting organisms and may be sufficient on its own [1,4,5]. Clinical use of local aminoglycosides began over 40 years ago, initially delivered with non-absorbable beads made of polymethylmethacrylate cement (PMMA) [2,6]. Walenkamp [7] popularized their use in the management of chronic osteomyelitis, but more recently, absorbable carriers (mainly inorganic salts and ceramics) have been used in clinical series managing osteomyelitis and fracture-related infections [8,9,10,11,12]. These studies have shown that local antibiotics can effectively fill dead spaces with high success rates. Rodham et al. [9] studied osteomyelitis after fracture and demonstrated that the recurrence rate was reduced from 17.6% to 9.8% with the use of local antibiotics (*p* = 0.01). Similarly, McNally et al., in a study of 433 FRIs, showed that treatment failed in 18.7% of those treated without local antimicrobials, but this reduced to 10% when local antibiotics (predominantly aminoglycoside alone) were used (hazard ratio 0.48; 95% confidence interval 0.29–0.81) [13].

Aminoglycosides, including gentamicin, display concentration-dependent killing, whilst vancomycin exhibits time-dependent killing [14,15]. Also, vancomycin is solely active against Gram-positive organisms, whereas aminoglycosides have a broader activity. These differences in mechanism of action may have important implications for the efficacy of these drugs when delivered locally, as a single implantation, into the infected bone. The relative efficacy of single-agent aminoglycoside versus combination local antibiotics has not previously been studied.

The rationale for this study was based on the principle that we should try to reduce unnecessary use of antimicrobials. No previous study has shown improved efficacy with the addition of glycopeptides to aminoglycoside alone in local therapy of bone and joint infection. In theory, adding vancomycin may increase the effectiveness against Gram-positive organisms which could be resistant to aminoglycoside. However, the very high levels of aminoglycoside achieved in local therapy may negate this potential advantage. In our previous study, local aminoglycoside alone was highly effective and laboratory-measured resistance to gentamicin did not affect outcome [12]. This retrospective study evaluated the effect of combination aminoglycoside and vancomycin local antibiotic treatment compared to aminoglycoside alone in the surgical management of bone infection. 

## 2. Results

### 2.1. Patient Demographics

A total of 1145 patients with bone and joint infections were reviewed. Of these, 441 received local antibiotic therapy; 266 had osteomyelitis or fracture-related infection, with local antimicrobial therapy and were included for analysis. After surgery, nine patients died from non-infectious causes before the outcome endpoint at 12 months and were excluded. Five patients were lost to follow-up and were excluded from analysis. A total of 252 patients were included in the final analysis, with a minimum of 12 months follow-up. 

In total, 113 patients had treatment with aminoglycoside alone (gentamicin 92; tobramycin 9, gentamicin and tobramycin 12), and 139 patients had a combination of aminoglycoside and vancomycin (vancomycin and gentamicin 109; vancomycin, gentamicin and tobramycin 30). 

Overall, the study cohort was a middle-aged population (median age of the aminoglycoside alone cohort was 57 years (interquartile range: 40–66) and in the combination aminoglycoside and vancomycin cohort was 52 years (interquartile range: 37–62)) with excision of osteomyelitis being the most common surgical intervention. Infection was present in 255 bones in 252 patients (98 tibia, 65 femur, 23 humerus, 17 fibula, 14 calcanei, 8 ulnae, 6 metatarsals, 5 radii, 5 ankle fusions, 4 pelvic bones, 3 wrists, 2 patellae, 2 hips, one each of phalanx, scapula and metacarpal). The most frequently isolated organism was *Staphylococcus aureus*, followed by non-*pseudomonas* Gram-negative organisms. Rates of confirmed gentamicin resistance and polymicrobial infection were similar between the two groups. The characteristics are presented in Table 1. 

### 2.2. Patient Outcomes

**Figure 1:**
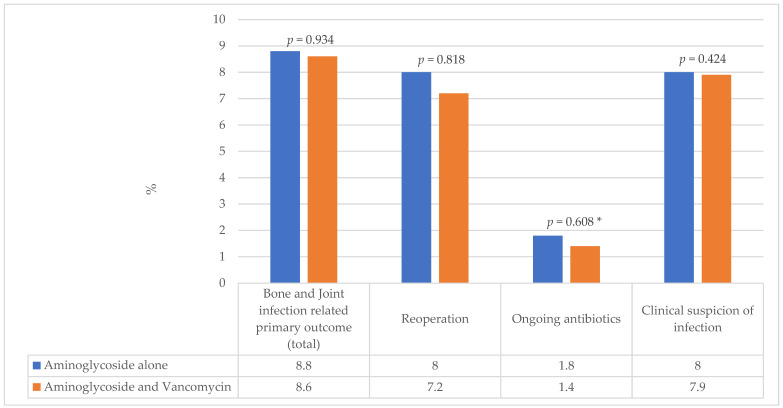
Percentage of cases identified with the Primary Outcome. * Fisher’s exact test used for cells with a count <5.

There was no difference in the rate of the primary outcome (failure to eradicate infection) between the two groups: 10/113 (8.8%) in the aminoglycoside alone and 12/139 (8.6%) in the combination group, *p* = 0.934 (Figure 1). There was no difference for reoperation, ongoing suppressive antibiotic use, or clinical suspicion of infection.

### 2.3. Multivariate Analysis

Bone Involvement, Anti-microbial options, Coverage of the soft tissues, Host status (BACH) score [16] showed similar rates of complex cases in both groups (68.8% amino only vs. 69% combined therapy), but the aminoglycoside only group had fewer uncomplicated cases (27.7% vs. 40%) and a small number of severely affected cases with limited treatment options (3.6% vs. 0%). The univariate associations between potential confounding variables and the primary outcome are presented in Table 2. Laboratory-confirmed aminoglycoside resistance was similar in both groups (10.6% vs. 10.1%), but presumed aminoglycoside resistance was more frequent in the aminoglycoside only group (23.9% vs. 15.8%). However, the presence of aminoglycoside resistance had no effect on failure rate (Odds ratio (OR) 0.39: 95% Confidence Interval (CI) 0.05–3.01, *p* = 0.37).

Multivariate analysis demonstrated that there was no added benefit of combination therapy (OR 1.54: 95% CI 0.59–4.04, *p* = 0.38). Higher BACH score and low BMI were associated with increased risk of failure (BACH OR 3.49: 95% CI 1.13–10.76, *p* = 0.03; BMI (per 1 unit increase) OR 0.91: 95% CI 0.84–0.99, *p* = 0.037). The presence of Gram-negative pathogens showed a trend towards an increased risk of failure (OR 2.35: 95% CI 0.96–5.71, *p* = 0.06).

### 2.4. Subgroup Analysis of Carrier Material Type

There were 229 patients who had CERAMENT as the sole carrier for local antibiotics or in combination with other bioabsorbable carriers (90 with aminoglycoside alone and 135 with aminoglycoside and vancomycin). There was no difference in the number of cases who met the primary outcome at 12 months; 9/90 (10.0%) with aminoglycoside alone versus 12/135 (8.89%) with combination therapy; *p* = 0.938.

Carriers can be implanted as injectable paste or pellets/beads or both. In this cohort, 148/252 (58.7%) patients had injectable paste only, of which 9/68 (13.2%) in the aminoglycoside alone group and 7/80 (8.75%) in the combination group met the primary outcome; *p* = 0.381. In addition, 45/113 (39.8%) aminoglycoside only and 59/139 (42.4%) aminoglycoside plus vancomycin patients had pellets or beads inserted; *p* = 0.674. 1/45 (2.2%) of the aminoglycoside only group and 5/59 (8.5%) of the combination group met the primary outcome at 12 months; *p* = 0.175. 

Comparing the form of the carrier material (injectable only versus injectable with pellets), there was no difference in the rate of the primary outcome (16/148; 10.8% injectable only vs. 6/104; 5.8% injectable plus pellets: *p* = 0.163).

## 3. Discussion

Local antibiotic delivery into an infected bone defect in osteomyelitis has become increasingly common in the treatment of bone and joint infection [11,12]. It results in high local concentration of antimicrobials and reduces concerns regarding patient compliance and systemic toxicity [17]. However, the choice of which empiric local antibiotic to insert into bone cement or void filler has not been investigated. In the past, gentamicin was the preferred antibiotic and was advocated in non-absorbable beads made from polymethylmethacrylate (PMMA) [2,18]. More recently, concerns about methicillin-resistant *Staphylococcus aureus* (MRSA) and reported increase in resistance to systemically administered aminoglycosides have prompted interest in additional local antimicrobials. However, it is important to limit the use of multiple antimicrobials without good evidence for additional efficacy, as part of good antibiotic stewardship. In particular, vancomycin is a valuable antibiotic for major Gram-positive infections and should be protected.

In this study, we found no overall advantage from adding vancomycin to gentamicin. This may be due to the delivery of sufficiently high gentamicin concentrations to overcome moderate resistance [19]. This is in keeping with a previous prospective clinical study at our institution which demonstrated that gentamicin-resistant organisms on laboratory testing (European Committee on Antimicrobial Susceptibility Testing (EUCAST) breakpoints) were successfully treated with local gentamicin [12].

Local antimicrobial therapy was provided using injectable paste and preformed pellets. We performed a sub-analysis to confirm that the form of the local antibiotic carrier did not affect the delivery of antimicrobial agent or the clinical outcome.

Ten percent of our patients had gentamicin-resistant isolates (EUCAST breakpoints); however, breakpoint definitions refer to systemic administration of antimicrobials [20]. Local antibiotics are able to achieve a far higher concentration than systemic antibiotics [2,19,21], with minimal risk of systemic toxicity [22]. CERAMENT G delivers 100 times the MIC for gentamicin-susceptible *Staphylococcus aureus* and *Pseudomonas* species [23], so may be able to overcome intermediate level antibiotic resistance. In vitro testing of CERAMENT G elution against *S. aureus*, *S. epidermidis*, *P. aeruginosa* and *E. cloacae* at different MICs demonstrated a zone of inhibition to all organisms excepting only highly resistant *S. aureus* (gentamicin MIC > 1024 mg/L) [24]. In vitro, gentamicin-loaded ceramic fillers are also able to inhibit some gentamicin-resistant coagulase negative *Staphylococci* [25]. 

Whilst local antibiotic treatment is generally safe, concerns remain for the potential of local toxicity on bone healing at the site of infection in in vitro studies [26,27], but this has not been confirmed in clinical studies. High-dose vancomycin and tobramycin may decrease cellular proliferation, but at standard doses in rabbit and rat models this effect was not seen [27], suggesting the importance of choosing an appropriate dose and class of local antibiotic. The method of delivery may also be important with modern bioabsorbable carriers providing a gradual elution of antimicrobial over several weeks [17,19].

### Limitations

This study is limited by its retrospective design with a relatively short follow-up period. Additionally, all patients received systemic antimicrobial therapy, and further studies into the effect of local antibiotic over and above standard systemic therapy are pending [28]. Depending on these results, a prospective study comparing gentamicin alone to combination gentamicin and vancomycin local therapy is required. 

This study is not a randomized controlled trial, so the results must be carefully interpreted. The study group was not selected for this therapy but was a consecutive series of cases presenting with the conditions to our department over the study period. All surgery was performed by the same surgical team (three surgeons) using the same protocol, with identical diagnostic criteria and systemic antimicrobial therapy (empiric and definitive). The choice of local antimicrobial(s) was not based on any previous microbiological culture results. Surgeons were free to choose whatever local antimicrobial regime they thought appropriate. The two groups were well matched with regard to many of the parameters which affect outcome. The factors which were different (BACH score, percentage of presumed gentamicin-resistant organisms and presence of Gram-negative organisms) all favored a better outcome in the combined vancomycin and aminoglycoside group, but this was not found. Clearly, the lack of randomization could introduce a selection bias, but we believe we have minimized this by the study design and open reporting of the full characteristics of the two study groups.

## 4. Materials and Methods

This was a retrospective observational cohort study at the Oxford University Hospital Bone Infection Unit. This specialist Unit treats a wide range of adult patients, referred from all over the United Kingdom, with infections affecting the musculoskeletal system, including infection after fractures, prosthetic joint replacement and hematogenous infection of the bones, joints and spine. 

### 4.1. Recruitment and Inclusion Criteria

The medical and surgical records and laboratory details of all patients who were referred between January 2019 and September 2022 were reviewed. We identified all consecutive patients treated with a diagnosis of hematogenous osteomyelitis and fracture-related infection. Patients who were under 18 years old at the time of treatment were excluded. Inclusion for outcome analysis required complete follow-up to a minimum of 12 months after surgery. Patients lost to follow-up or dying before 12 months were excluded from the analysis.

Infection was confirmed using the International FRI Consensus Definition [29]. Patients with infection of the spine, septic arthritis alone or infection following prosthetic joint replacement were excluded. Additionally, patients were only eligible for inclusion if five or more separate tissue samples were taken at surgery, for microbiological culture. If implants were removed at surgery, these could also be sent for culture of fluid after sonication [30], but this was not mandatory. The culture protocol has previously been reported [31]. Infection was confirmed with at least two positive cultures of a phenotypically identical organism. A single positive culture was accepted when there was also confirmatory evidence of infection on histopathology. Tissue samples for histology (usually 3 or more) were embedded in paraffin and 5 µm sections cut. Sections were stained with hematoxylin and eosin and Gram-stained. At least 10 high-power fields were examined per section (×400). Positive histology was defined as the presence of visible micro-organisms on Gram stain or an average of ≥5 polymorphonuclear neutrophils seen per high-power field on hematoxylin- and eosin-stained sections. 

Patients were only included if they had implantation of antibiotic carriers delivering aminoglycoside, with or without vancomycin, as part of their surgical management. 

### 4.2. Surgical and Medical Treatment

**Figure 2:**
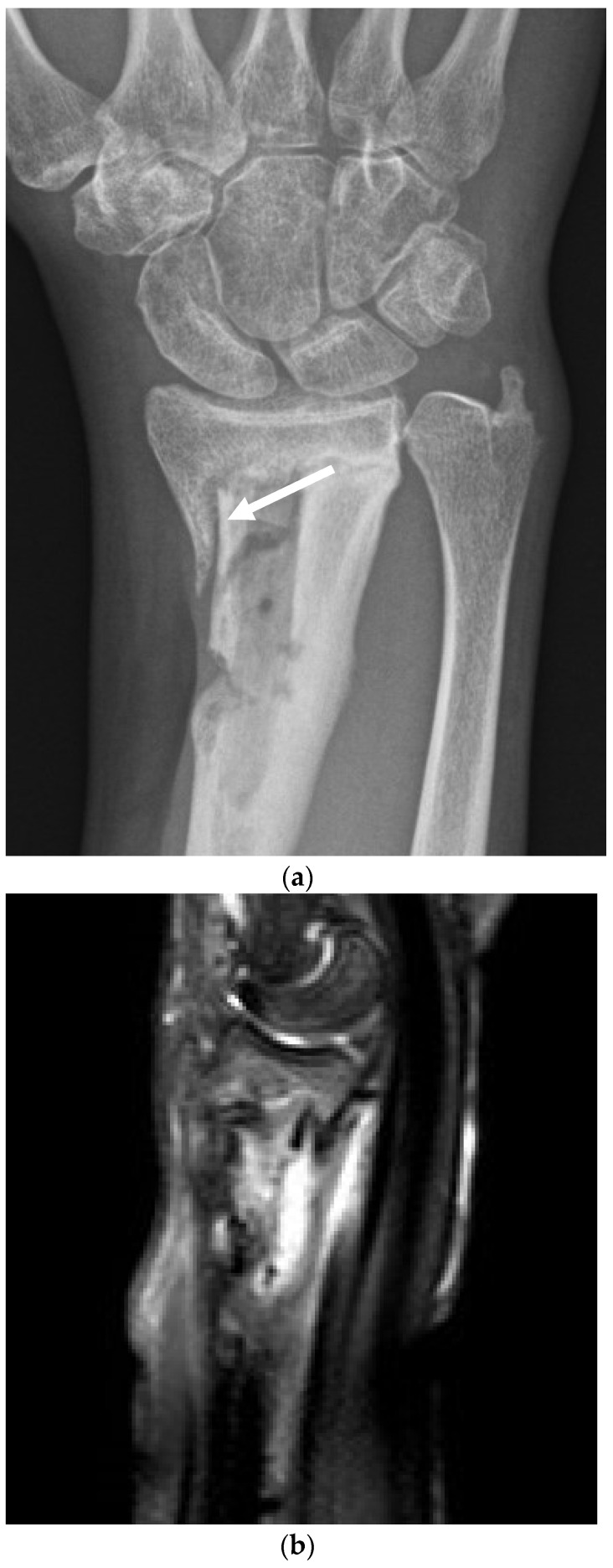

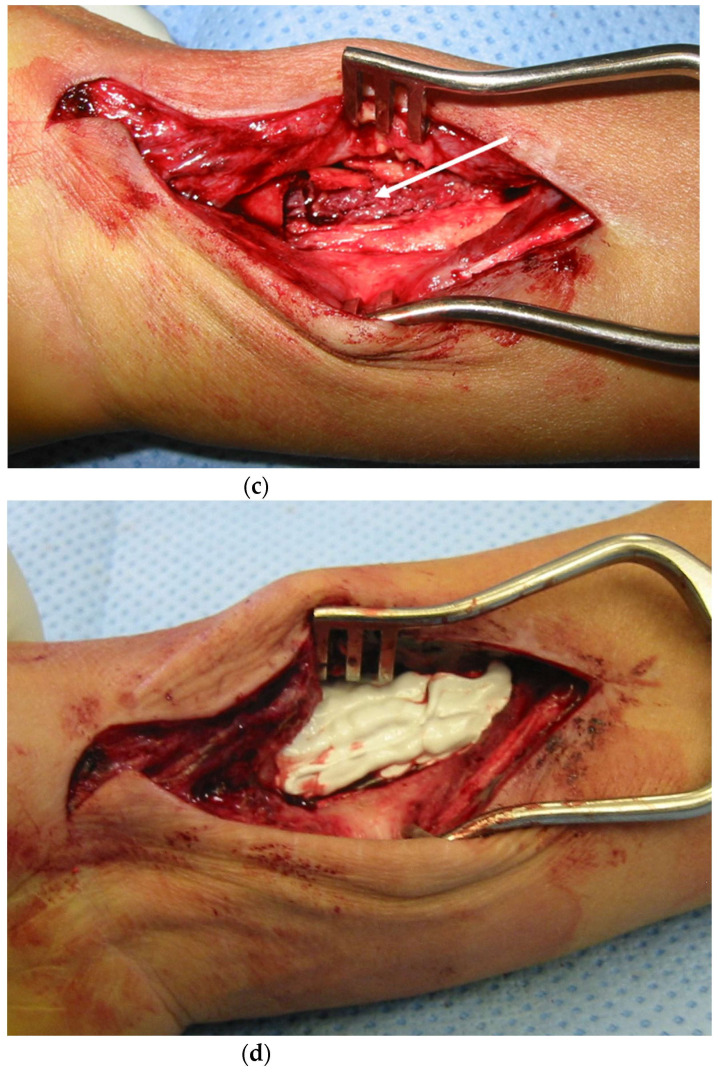

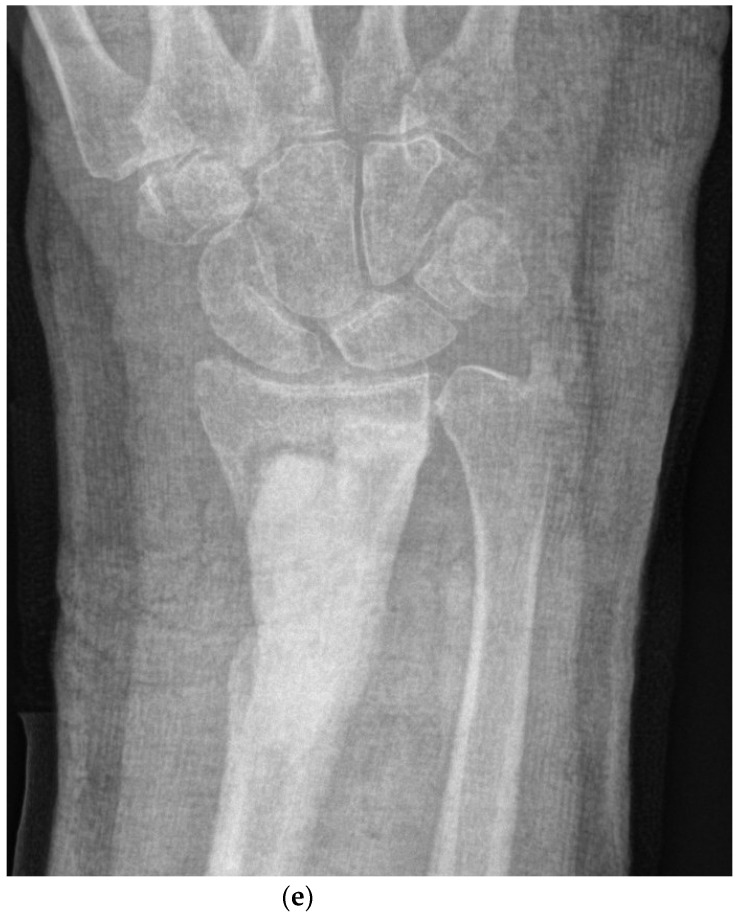
(**a**). The surgical management with implantation of a gentamicin-loaded antibiotic carrier. This 32-year-old woman suffered a closed fracture of her distal radius in a fall. After fixation with a plate, the fracture became infected. The plate was removed, but she continued with a draining sinus and active infection. This preoperative radiograph shows the dead bone in the radial metaphysis (white arrow), but the fracture has healed. (**b**). The magnetic resonance scan demonstrates the area of high signal around the dead bone, with active infection. (**c**). At operation, the volar aspect of the wrist was opened and the infected bone exposed. Five deep tissue samples were taken for microbiology and three for histology. The central area of necrotic bone and infected tissue (white arrow) was excised back to healthy bleeding bone. (**d**). The excised dead space has been filled with CERAMENT G (with gentamicin), completely filling the bone defect. The material was compressed into the bone and the skin closed in layers. (**e**). The postoperative radiograph shows the antibiotic carrier in place, filling the defect.

All patients were treated with a single-stage protocol incorporating deep tissue sampling, excision of dead and compromised tissue, management of the excised dead space, bone stabilization (if required) and soft tissue closure, as previously described [32] (Figure 2a–e). 

Briefly, antibiotics were stopped at least two weeks before surgery when possible (stable patient without fever). Surgery was performed under tourniquet where possible. Sinus tracts and unstable skin were excised and discarded. Deep samples were taken by a validated protocol [33] involving harvest of at least 5 specimens of tissue, each taken with clean instruments and avoiding contact with the skin. Samples were transferred immediately to the microbiology laboratory for processing as described above. At least 3 specimens were also taken for histopathology [34].

All dead tissue was excised from the infected zone, preserving the peripheral living tissue. This was performed with gouges and chisels. Small spaces were excised with a saline-cooled bone burr, and screw holes were over-drilled. If the bone was unstable (unhealed fracture, non-union or very thin bone after resection), stabilization was performed, usually with the application of an external fixator.

In all cases, the dead space was filled with local antibiotics delivered in a carrier material. No patient had antibiotic powder implanted alone. The form of the carrier (injectable paste or beads or pellets) was chosen depending on the morphology of the space to allow good void filling. At the time of this study, there was no published evidence-based recommendation concerning the sole use of aminoglycoside or combined antibiotic use in local therapy for bone infection [27]. Multiple licensed commercial products were available (see Table 1) with various options for aminoglycoside or combined therapy. In this study, we defined two groups of patients by the type of antimicrobial(s) delivered locally into the bone. Group one had only an aminoglycoside antibiotic (gentamicin or tobramycin) delivered in a licensed carrier material. Group two also had aminoglycoside but in combination with vancomycin; both delivered in a licensed carrier material. Surgeons were free to choose their preferred material and antibiotic(s). This choice had no effect on any other aspect of the surgical or medical care. Antimicrobial choice was not based on any microbiological information obtained before surgery, as this was not regarded as relevant to local therapy.

After dead space filling, the soft tissues were closed during the same operation. No vacuum-assisted closure or delayed closure was employed. When the defect was too large for direct skin apposition, a local or free tissue transfer was performed to provide immediate skin coverage.

Empiric parenteral antibiotics were given after sampling and continued until microbial cultures were obtained. The regimen was chosen based on our previously published study of osteomyelitis and FRI [35]. The regimen was intravenous administration of 1–2 g of vancomycin (weight-dependent) and 500 mg of meropenem after sampling. Vancomycin was continued twice daily and meropenem 3 times daily for two days. If no Gram-negative organisms were cultured by the third day, meropenem was topped and vancomycin continued. When definitive microbiological cultures were obtained (usually within 5 days), the long-term antimicrobial therapy was decided. Patients were preferentially treated with oral therapy, when an oral option was available, based on the results of the OVIVA randomized trial [36]. The individual choice of drug was consistent with the recommendations of the FRI consensus guidelines [37] and continued for 6–12 weeks. 

Patients were followed up for a minimum of one year or to death, if sooner. Patients visited a specialist combined clinic (with surgeons and infectious disease physicians), usually at 6, 12, 26 and 52 weeks after surgery. Patients with external fixators attended more frequently for review of fixator pin sites and stability. On each visit, the primary outcome was assessed with clinical assessment, radiology and blood tests when indicated. 

### 4.3. Data Collection

Medical records were reviewed for inclusion and exclusion criteria, and data were extracted using a standardized template. Demographic information collected included age, gender, Body Mass Index (BMI) and American Society of Anesthesiology (ASA) score [38]. Clinical data collected included BACH classification [16,39], microbiological characteristics including antimicrobial susceptibility profile, type of local antibiotic carrier, duration of antibiotic therapy and outcome. Presumed gentamicin resistance was based on the Sanford Guide antibiogram [40].

### 4.4. Outcome

The primary outcome of failure of therapy was a composite of recurrence of infection, continued or new antimicrobial therapy, or reoperation with suspected or confirmed infection at one year after index surgery [28]. Any patient presenting with any one or more of the components of this composite, by one year of surgery, was regarded as having failed therapy. Subgroup analysis of the type and form of carrier material used was also performed.

### 4.5. Data Management and Analysis

Analysis was performed using IBM SPSS v29. The difference in the rate of the primary outcome between the two groups was compared using logistic regression. Potential confounders considered for inclusion in the regression models were age, ASA, BMI, presence of gentamicin resistance, type of surgery, presence of polymicrobial infection, presence of Gram-negative organisms, BACH score, time from intravenous to oral antibiotic switch and duration of antibiotics. Each potential confounder was examined separately by univariate analysis to determine their association with the outcome variable. Confounders whose association with the outcome variable had a *p*-value < 0.2 were included in the multivariate model, except for age which was included regardless.

## 5. Conclusions

Clinical outcome was not improved by the addition of vancomycin to aminoglycoside alone as local therapy for the management of osteomyelitis and fracture-related infection. Additionally, laboratory-measured resistance, using currently accepted EUCAST breakpoints, may not be relevant in local therapy. This study should have an impact on the choice of empirical local antibiotic treatment used in the treatment of bone and joint infections.

## Figures and Tables

**Table 1:** Patient Demographics.

Characteristics	Aminoglycoside Alone		Aminoglycoside and Vancomycin	
	Median	IQR *	Median	IQR *
Age (years)	57	40–66	52	37–62
ASA Score *	2	1–3	2	1–3
BMI *	26	22–30	28	24–32
	**n**	**%**	**n**	**%**
Total	**113**	**100**	**139**	**100**
Male	85	75.2	102	73.4
*Type of Surgery*				
Osteomyelitis excision	69	61.1	87	62.6
Fracture-related infection with hardware removal	38	33.6	44	31.7
Fracture-related infection with hardware removal and reimplantation	6	5.3	8	5.8
*BACH score* *				
Uncomplicated	32	28.3	55	39.6
Complex	77	68.1	84	60.4
Limited options	4	3.5	0	0
*Local antibiotic carrier* ^§^				
CERAMENT alone	68	60.2	80	57.6
OSTEOSET T only	9	8	0	0
Herafill G only	9	8	0	0
CERAMENT with Herafill G	9	8	24	17.3
CERAMENT and OSTEOSET T	13	11.5	30	21.6
Refobacin G	4	3.5	0	0
Stimulan	0	0	4	2.9
CERAMENT, Herafill G and Septopal G	0	0	1	0.7
Herafill G and Septopal G	1	0.9	0	0
**Post-surgery characteristics**	**Median**	**IQR**	**Median**	**IQR**
Duration of antibiotics (days)	42	42–42	42	38.5–42
IV * to oral switch (days)	5	4–6	5	4–6
	**n**	**%**	**n**	**%**
Organism				
*Staphylococcus aureus*	53	46.9	92	66.2
Coagulase negative *Staphylococcus*	35	31.0	28	20.1
*Streptococcus* species	21	18.6	25	18.0
*Pseudomonas* species	11	9.7	7	5.0
Other Gram-negative organisms	42	37.2	30	21.6
Other Gram-positive organisms	29	25.7	17	12.2
Polymicrobial infection	38	33.6	44	31.7
Confirmed gentamicin resistance	12	10.6	14	10.1
Presumed gentamicin resistance	27	23.9	22	15.8

* IQR: Interquartile range, ASA: American Society of Anesthesiology, BMI: Body Mass Index, BACH: Bone Involvement, Anti-microbial options, Coverage of the soft tissues, Host status, IV: intravenous. ^§^ CERAMENT G and V, Bonesupport AB, Lund, Sweden. OSTEOSET T, Wright Medical, Arlington, VA, USA. Herafill G, Heraeus Medical GmbH, Wehrheim, Germany. Refobacin G, Zimmer Biomet, Warsaw, IN, USA. Stimulan, Biocomposites, Keele, UK. Septopal G, Zimmer Biomet, Warsaw, IN, USA. CERAMENT and Stimulan can be implanted with gentamicin and/or vancomycin. OSTEOSET T, Herafill G, Refobacin G and Septopal G only deliver aminoglycoside.

**Table 2:** Univariate analysis of association with the primary outcome.

Outcome	Category	OR	95% CI	*p* Value
Primary Outcome	ASA (per 1 unit increase)	1.05	0.61–1.82	0.86
	Age (per 1 year increase)	1.00	0.98–1.03	0.76
	BMI (per 1 unit increase)	0.94	0.86–1.02	0.11
	Confirmed and presumed gentamicin resistance	0.39	0.05–3.01	0.37
	Type of surgery: osteomyelitis resection	0.88	0.35–2.19	0.79
	Presence of polymicrobial infection	1.51	0.62–3.69	0.37
	Presence of Gram-negative organism	2.35	0.96–5.71	0.06
	BACH score (per 1 unit increase)	3.29	1.12–9.23	0.02
	IV to oral switch (per 1 day increase)	0.95	0.82–1.11	0.53
	Duration of antibiotics (per each week increase)	1.00	0.99–1.01	0.94

OR: odds Ratio, ASA: American Society of Anesthesiology, BMI: Body Mass Index, BACH: Bone Involvement, Anti-microbial options, Coverage of the soft tissues, Host status, IV; Intravenous.

## Data Availability

The data presented in this study are available upon request from the corresponding author due to privacy reasons.
